# Plastic Waste in Latin America and the Caribbean (LAC): Impact on the Environment and Public Health—A Systematic Review

**DOI:** 10.1155/2024/5698516

**Published:** 2024-09-30

**Authors:** Danladi Chiroma Husaini, Rodeli Kaylin Mendez, Michael Arzu, Lydia Harris-Thurton

**Affiliations:** Allied Health Department Pharmacy Program Faculty of Health Sciences University of Belize Belmopan Central Campus, Belmopan, Belize

## Abstract

**Background:**

The global spread and accumulation of plastics in freshwater, marine, and terrestrial settings are of great concern to public health and the environment, especially in developing countries with few resources. In the Caribbean and Latin America, nearly 17,000 tons of plastic waste are generated and trashed daily in open dumpsites with attendant consequences for the environment, the economy, aquatic life, the beauty of sea beaches, and public health. The increased use of plastics threatens public health and the ecosystem. *Main Body*. This systematic review assessed the impact of plastic waste on the environment, economy, and public health in LAC by searching relevant databases such as PubMed, HINARI, Google Scholar, and Scopus. PRISMA and Rayyan software were used to select and analyze research articles for the review.

**Conclusions:**

The review showed that plastic pollution significantly impacts the environment, aquatic life, economy, and human health in LAC. The review further indicated that countries in LAC are working assiduously to address the issues associated with plastic pollution. The use of biodegradable plastics, cleanup campaigns, and policies/programs to reduce or ban plastics are some current efforts in many LAC countries. More research on the impact of plastic waste needs to be conducted, especially in the Caribbean, to address and mitigate the challenges of plastic pollution.

## 1. Introduction

The extensive use and application of plastics have led to increased production, especially with innovative polymer technologies from petrochemicals. Over the last 65 years, plastic products have contributed to an improved quality of life; however, this has increased their use, production, and waste [1]. Because of their low weight, durability, and low cost compared to other materials such as metals, plastics find diverse applications, leading to an escalation in public and environmental health issues, especially in developing countries with fewer resources and inadequate waste disposal measures.

Currently, a few strategies, such as microbial degradation of plastics and conversion to other valuable materials, incineration, biodegradable plastics, landfilling, and recycling, have been adopted to address the issues of plastic pollution [2]. However, most of these strategies are yet to address the vast amount of plastic waste generated in Latin America and the Caribbean (LAC), with many countries yet to adopt effective measures to address the threat of plastic pollution.

LAC countries are particularly affected by an epidemic of pollution by their inability to deal with plastic waste due to inadequate resources, weak regulatory policies, and inefficient garbage collection and disposal measures. Regarding plastic pollution, nearly 17,000 tons of plastic waste are generated and disposed of daily in open dumpsites in Latin America and the Caribbean (LAC). The low-income nature of the region, rising tourism, high incidence of violent crime and corruption, and close to 40 million people lacking access to essential waste collection services make the region vulnerable to environmental pollution from plastic waste [3–6]. This systematic review examines LAC studies on plastic waste and its environmental and public health implications. The review synthesizes current research on plastic waste in LAC, assesses the research progress in plastic waste, and provides a direction for the future within the LAC region.

## 2. Main Text

### 2.1. Review Question

What are plastic waste's environmental and public health impacts in the Caribbean and Latin America?

### 2.2. Methodology

Database search strategy exclusion and inclusion criteria: PubMed and EBSCOhost databases were searched for articles on plastic waste and pollution from 2012 to April 2022. The search keywords and terms included “plastics,” “plastic waste,” “plastic pollution,” “environmental pollutants,” “impact of plastic waste,” “Latin America,” and “the Caribbean.” Records found were managed using the Endnote library.

### 2.3. Articles Screening

Two investigators reviewed the article's title, abstracts, and full text to eliminate irrelevant duplicate records in line with the study characteristics (Table 1). An investigator supervised the entire screening process to ensure the assessment and inclusion of quality records and to minimize the risk of bias. Only studies on plastic waste and its impact on environmental and public health conducted in Latin America and the Caribbean were eligible for inclusion in the systematic review. PRISMA and Rayyan systematic review software were used to screen and analyze articles. The risk of bias potential for each included study was checked using the Revised Cochrane risk-of-bias tool for randomized trials (RoB 2).

### 2.4. Data Abstraction

Two analysts independently extracted applicable information from the eligible articles. Each selected article was assessed based on the quality criteria defined to address the issues of plastic waste and its implications for the economy, environment, and public health in Latin America and the Caribbean. Relevant information such as study design, methodology, year of publication, country of study, data collection period, and study findings was extracted and entered into an Excel spreadsheet for synthesis and analysis. A meta-analysis was not conducted.

### 2.5. Risk of Bias

The reviewers working independently minimized the potential risk of bias and had the “Blind On” feature in Rayyan. The RoB 2 was downloaded in an interactive Excel sheet, and the five domains each had a low risk. The domains are the randomization process, missing outcome data, deviations from the intended interventions, outcome measurement, and imported result selection.

### 2.6. General Findings

**Figure 1:**
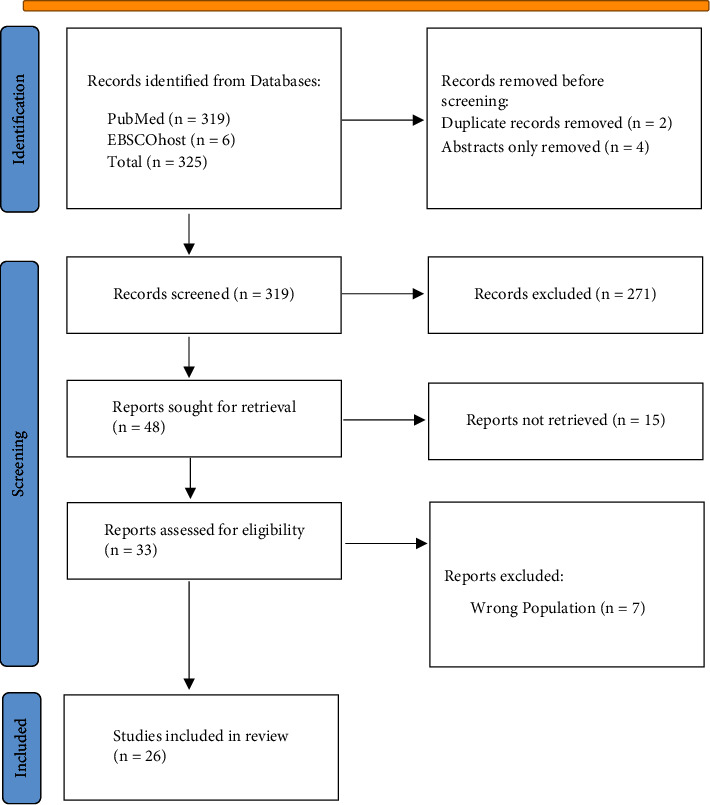
PRISMA flowchart for study identification and selection process.

Figure 1 presents this systematic study's database identification and article selection process. A total of 325 articles were found, out of which duplications (*n* = 2), abstracts only (*n* = 4), unrelated titles (*n* = 271), full articles not accessed (*n* = 15), and wrong population (*n* = 7) were removed and excluded from the study. Twenty-six studies were eligible and eventually included in the systematic review (Figure 1).  Table 2 summarizes findings on plastic waste in Latin America and the Caribbean. Finally, Table 3 presents suggestions on how to address plastic pollution in LAC.

The Pacific Coast of Columbia articles represented most in this review (Table 2). These studies were identified and utilized for this review [9–12]. Plastic wastes identified in the article included microplastics, macroplastics, and anthropogenic waste. Sources of plastic waste pollution were identified as illegal dumping [7], land and marine debris [8, 15–17], deep-sea litter [13], marine litter [16, 17, 19], land [10], and urban sources [18]. The studies attributed the causes of plastic waste pollution to poor waste management, limited recycling, and weak enforcement [7, 9]. In addition, humans and tourists also contribute to plastic waste litter (Table 2) [9, 14, 17].

The implications of generated plastic waste on the environment, health, and economy were also addressed in the study. For instance, the Caribbean dependence on the marine ecosystem [7], tourism, and fishing [14] were highlighted. Hence, there is a need to protect biodiversity [8] from threats to the coastal and marine ecosystem [9, 12]. Social and economic wellbeing [7] can be addressed through education campaigns (Table 2) [7, 9].

The policies addressed in the study include banning single-use plastic and polystyrene and having fines and penalties for noncompliance [7, 18]. There was an emphasis on prevention, mitigation, and control to mitigate the leakage of plastic waste in the marine ecosystem [8, 9, 14, 16]. Scientific research could be conducted to inform policies [12, 13, 18]. Debris categorization and particle tracking are vital for future research (Table 2) [8].

### 2.7. Impact of Plastic Waste in Latin America and the Caribbean

Plastic waste entering the environment impacts the public health and the beauty of ocean beaches and can harm aquatic animals through ingestion or entanglement. In addition, plastic wastes provide breeding environments for disease-causing vectors such as mosquitoes, affect wastewater systems, and block drainage, leading to increased flooding [21, 26–28]. Furthermore, microplastics are now reported in the human food chain, increasing public health risk [29, 30]. Recently, Zheng and Suh [31] reported that greenhouse emissions result from the production, use, collection, and disposal of plastic products in the environment. The systematic review discussed microplastics, macroplastics, and COVID-19, the economic challenges of plastic waste, and highlighted possible solutions to address the epidemic of plastic pollution.

### 2.8. Microplastics

The plastic debris is categorized as macroplastics (>5 mm) and microplastics (<5 mm). This review showed that Latin America and the Caribbean regions contributed to 5% of the global scientific output on microplastics, and overall, the highest contributor within the region was Brazil (52%), followed by Chile (16%) and Mexico (13%). For instance, 46 species from the Amazon River on the North Coast of Brazil were analyzed, with 30% of the species having ingested microplastic particles, mostly pellets [32]. Planktons are an essential food source for various fish species. Hence, microplastics floating in coastal waters are prone to be ingested in large amounts by planktons when they confuse them for food with the possibility of transferring them into the food chain [33–35]. Furthermore, the stomachs of nineteen deep-water butterflyfish collected from St. Peter and St. Paul's Archipelago in Brazil were analyzed for microplastics, with 6 of the butterflyfish found to contain microplastics [36]. Consequently, fish and other marine species living in coastal areas are more likely to ingest microplastics, increasing the chances of human microplastic ingestion and leading to public health challenges [37, 38]. Behavioral abnormalities, growth retardation, and neurotoxicity have been reported in fish due to microplastics (MPs) exposure. Moreover, immunomodulation, neurotoxicity, cytotoxicity, and oxidative stress are possible consequences of human exposure to MPs, with inhalation, consumption, and skin contact as the main routes of exposure to environmental MPs [39].

### 2.9. Macroplastics Anthropogenic

Plastic waste, also called plastic pollution, is the assemblage of plastic substances in the environment that affects wildlife, their habitats, and humans. Macroplastics are plastic debris larger than 5 mm (>0.5 mm). Anthropogenic waste also refers to marine debris from human-generated objects. Macroplastics are introduced into the environment through terrestrial, aquatic, and atmospheric means. Many factors contribute to anthropogenic waste. For example, the spatial distribution of plastics was reported to be due to the position of the beach relative to wind and currents [40]. Although plastic straws account for a fraction of city residue, they also pollute coastal waste and marine environments, raising concerns in America and leading to policies, laws, and actions to curb or prohibit inappropriate disposal [41]. Public health and aquatic life are negatively impacted by macroplastics in the environment. In aquatic ecosystems, ingestion and entanglement of aquatic life are significant threats to aquatic life [42].

### 2.10. Plastic Waste during the COVID-19 Pandemic

Although plastic waste has been an ongoing global problem, the COVID-19 pandemic added to the global burden of plastic waste and is reported to be an emerging threat to LAC [43, 44]. Recent reports have indicated that more than eight million tons of pandemic-associated plastic waste have been generated globally, with most coming from hospitals [45]. This pandemic has substantially increased the use and production of face masks and other protective components (protective suits, gloves, face protectors, and safety shoes) manufactured with polymeric materials. The release into waterways of nanofibers with engineered nanoparticles (ENPs) from antiviral face masks during wash cycles in washing machines and the indiscriminate use of commercial disinfectant products with ENPs could potentiate the negative impact of microplastics (MPs) pollution on some marine species. Moreover, 80% of global tourism occurs in coastal areas; therefore, the pollution generated by this type of activity quickly reaches beaches and seas, thus polluting aquatic life [46]. Mass tourism promotes an occupation of the coastal area, capable of generating a high impact, transforming or degrading landscapes and ecosystems. Focusing on South America, using and mismanaging personal protective equipment (PPE) represents an environmental problem. Added to this issue are the rise in single-use plastic utilization and the decline of plastic recycling due to the restrictions generated by the pandemic, further heightening plastic pollution on coasts and beaches [47]. Therefore, this will continue as a cycle until unnecessary plastic materials are reduced.

### 2.11. Economic Challenges of Plastic Waste

The United Nations Environment Program documented that the economic damage to ecosystems and marine life from plastic pollution costs an estimated USD 13 billion annually. The cost to clean beaches polluted with plastic waste, tourism activities, and losses to the fishing industry made up part of the cost of plastic pollution [48]. Most countries in LAC were facing varying economic challenges before the COVID-19 pandemic. The pandemic (COVID-19) added to the already existing challenges, such as the epidemic of plastic pollution in many LAC countries. Plastic pollution has several adverse effects on locally generated economies by reducing tourists and losing tourism incomes due to marine litter [49]. For instance, because most Caribbean countries depend mainly on a healthy marine ecosystem as a significant source of income, the Caribbean countries are reported to generate and dispose of a substantial amount of plastic waste, making it the top 10 global marine polluters per capita [1, 7]. Therefore, for the economy of these countries to develop, there must be laws in place that will protect the coastal areas from plastic pollution so that tourists can be attracted to a clean and healthy environment.

### 2.12. Solutions to Reduce Plastic Waste in Latin America and the Caribbean

Social settings, geographical location, cultural practices, and local, national, or regional policies impact the cost-effectiveness of managing plastic pollution. A few intervention models, such as reuse, recycling, replacement of plastics with alternative products, postconsumption management strategies, and outright banning of plastics, have been proposed to reduce the burden of plastic waste (Table 3) [21]. In many countries, nongovernmental organizations and local and scientific communities work assiduously to address plastic pollution challenges. Although significant awareness is being raised, strategic, practical, and measurable interventions are still required to effectively minimize the menace of plastic pollution on public health and marine life.

Furthermore, regulatory (e.g., bans) or economic (e.g., levies) instruments should be introduced in stages and move forward in phases, with education campaigns and good marketing of alternatives. Adequate lead time (i.e., more than a year) would help, mainly if supported by research and development. This approach is closer to the model used in Antigua and Barbuda. Penalties are usually necessary to deal with recalcitrant operators, but in many cases, a system of phased substitution, with adequate alternatives available, would make the process far more manageable. The battle is won once the public is persuaded of the case for switching. Economic instruments such as regulation of production, levies, or taxes can also contribute to developing more sustainable alternatives.

The issue of plastic pollution has grown to the point where approximately 175 countries have agreed to develop a global treaty that addresses plastic waste and present a resolution. This treaty would be the first of its kind; however, it has been one of the many actions that countries have taken to attempt to deal with plastic pollution. The Inter-American Development Bank (IDB) recently launched an Open Innovation Challenge to find creative ways to minimize or eliminate single-use plastic and plastic waste in Latin America and the Caribbean. The IDB initiative indicated that ten finalists will be invited to its annual meeting in Colombia in March 2020 and that the challenge offers USD 60,000 in cash prizes, with a top prize of US $30,000 [50]. Other countries have adopted various hybrid approaches to address plastic pollution challenges within the region, with relative successes recorded [51].

Currently, many LAC countries are enacting policies and programs to regulate the use of plastics and plastic pollution. The countries are using innovative technology, bans, and taxes to minimize the generation and consumption of plastics to reduce the impact on public health, marine life, and oceans [52]. As of 2021, 12 countries in the Caribbean have banned the use and import of plastics and styrofoam. These countries include the Bahamas, St. Lucia, Antigua and Barbuda, Barbados, Jamaica, Belize, the Dominican Republic, Grenada, Guyana, St. Eustatius, St. Vincent and the Grenadines, as well as Trinidad and Tobago [53]. Furthermore, in Latin America, countries such as Mexico, Argentina, Ecuador, Brazil, Chile, Colombia, Costa Rica, and Panama have banned single-use plastics or have made projections to ban the use of plastics soon [52]. In addition, Barbados, Brazil, Chile, Colombia, Costa Rica, Grenada, Ecuador, Panama, Peru, Dominican Republic, Saint Lucia, and Uruguay are some of the countries in LAC that have signed up for the campaign by the United Nations Environment for clean seas [52].

### 2.13. Limitations

This systematic review has a few limitations. The inability of the reviewers to access articles that were outside the English language was a limitation, mainly because some countries in LAC are non-English speakers. Moreover, the screening process of the articles could have been more robust, and the diversity of the locations and authors would have strengthened the review. In addition, only a few studies were found in LAC, especially in the Caribbean, thereby limiting the averseness of the review. Finally, articles on land plastic pollution were not accessed, and a meta-analysis needed to be conducted. Despite these limitations, this systematic review has its strengths. The quality of the research articles was assessed using the CASP-Qualitative tool checklist, thereby ascertaining the appropriate inclusion of articles that address the subject matter. The gaps identified in the review, such as preliminary research and publications on plastic pollution, stimulate the scientific community to be more proactive in plastic pollution research.

## 3. Conclusions

In order to ensure the preservation of public health, the environment, and sustainable economies in the use of plastics, long-term interventions such as pre- and postconsumption regulation of plastics should be explored. Sustainable substitution for plastics and biodegradable materials should be recommended and utilized at all levels. Drastic reduction or minimization of unnecessary plastics and recycling using mechanical or chemical conversions of plastics should be highly encouraged in every LAC country. The reduction or minimization of postcollection leakage through incineration and landfilling should be considered, while policies should be enacted, implemented, and enforced for plastic use. At every level, governments, industry, researchers, and communities must provide appropriate engagement through education and support toward reducing plastic pollution in Latin America and the Caribbean.

## Figures and Tables

**Table 1:** Inclusion and exclusion criteria.

Inclusion	Exclusion
Population: Latin America and the Caribbean	Articles from countries outside Latin America and the Caribbean
Language: English	Language: non-English articles
Plastic waste management	Duplications or overlapping data
Effects of plastic waste on wildlife	
Effects of plastic waste on human health	
Causes of plastic waste	
Conditions that result from plastic waste	
No restriction on age, race, gender	
Macro, micro, meso, and nano plastics	
Types of articles: published	Unpublished articles
Articles from 2012–April 2022	Articles before 2012 or after April 2022

**Table 2:** Plastic waste in Latin America and the Caribbean.

Outcomes	Impacts	Number of studies
Location	(1). Caribbean [7, 8] (2). Caribbean and Pacific Coast of Columbia [9–12] (3). Southern Caribbean [13, 14] (4). Guatemala [15] (5). Marine protected area [16] (6). Central America [Belize] [17, 18] (7). Bahamas [19]	13

Plastic waste (macroplastic, microplastic, anthropogenic)	(1). Illegal dumping [7] (2). Land and marine debris [8, 15–17] (3). Deep sea litter [13] (4). Marine litter [16, 17, 19] (5). Land [10] (6). Urban source [18]	13

Implications on the economy, public health, and the environment	(1). The Caribbean depends on a marine ecosystem [7] (2). Tourism and fishing [14] (3). Social and economic well-being [7] (4). Education campaigns [7, 9] (5). Biodiversity [8] (6). Threat to the coastal and marine ecosystems [9, 11]	6

Sources of plastic waste	(1). Poor waste management, limited recycling, weak enforcement [7, 9] (2). Tourism [9, 14] (3). Humans [17]	5

Policies on plastic waste	(1). Banned single-use plastic and polystyrene; fines and penalties for noncompliance [7, 18] (2). Prevention, mitigation, and control [7, 14, 16] (3). Debris categorization and particle tracking [8] (4). Mitigate leakage of plastic waste in the marine ecosystem [8] (5). Scientific research [12, 13, 18]	10

**Table 3:** Solutions to reducing plastic pollution.

References	Plastic wastes intervention	Description	Example
[20, 21]	Substitution	Substitute plastics with an alternate material	The use of bio-based and biodegradable materials, glass, paper
[21–23]	Reduction	Eliminate or reduce plastics	Eliminate single-use plastics, reuse plastics, eliminate unnecessary uses of plastics, use of compostable materials
[21, 23, 24]	Recycling	Mechanical and chemical conversion of plastics, reuse of plastics	Reduce exports of plastics, improve collection and sorting of plastics, improve conversion capacity, increase the amount of recycling, reuse of plastics
[21, 23]	Disposal	Minimize or reduce postcollection leakage	Landfilling, incineration
[21, 25]	Policies	Enact, educate, implement, and enforce policies on plastics	Government policies, educational campaigns, community engagements, industry commitments

## Data Availability

The data used to support the findings of this study are available from the corresponding author upon reasonable request.

## Abbreviations

CASP: Critical appraisal skill program
COVID-19: Coronavirus 2019
ENP: Engineered nanoparticles
EPA: Environmental protection agency
IDB: Inter-American Development Bank
LAC: Latin America and the Caribbean
NEPA: National Environmental Policy Act
NGO: Nongovernmental Organization
PPE: Personal protective equipment
PRISMA: Preferred reporting items for systematic reviews and meta-analyses
SUPs: Single-use plastics
UNEP: United Nations Environmental Program
UNWTO: United Nations World Tourism Organization
USD: United States dollars
WHO: World Health Organization.
